# Supplementary material for: Impact of depression on stroke outcomes among stroke survivors: Systematic review and meta-analysis
Source: PLoS One. 2023 Dec 1;18(12):e0294668. doi: 10.1371/journal.pone.0294668 (PMC10691726; doi:10.1371/journal.pone.0294668)
Supplement: S1 File — (DOCX) [file pone.0294668.s001.docx]

**Impact of depression on stroke outcomes among stroke survivors: systematic review and meta-analysis.**

**Terms**

1. **Depression**

**Free text –** depression OR "major depressive disorder" OR "Depressive Disorder" OR "common mental disorder" OR "mental disorder" OR "mental health" OR "psychological morbidity" OR "depressive episode" OR "persistent depressive disorder" OR "major depressive disorder" OR "mood" OR "emotional distress"

**Mesh term –** "Depression"[Mesh] OR "Depressive Disorder"[Mesh] OR "Depressive Disorder, Major"[Mesh]) OR (“Major Depressive Disorder 1" [Supplementary Concept] OR "Major Depressive Disorder 2" [Supplementary Concept]

1. **Stroke**

**Free text –** stroke OR "hemorrhage stroke" OR "ischemic stroke" OR "transit ischemic stroke"

**Mesh term –** "Stroke"[Mesh] OR "Stroke, Lacunar"[Mesh] OR "Infarction, Posterior Cerebral Artery"[Mesh] OR "Brain Stem Infarctions"[Mesh] OR "Infarction, Middle Cerebral Artery"[Mesh] OR "Infarction, Anterior Cerebral Artery"[Mesh]

1. **Outcome**

**Free text –** outcome OR result OR "end-result" OR product OR rehabilitation OR recovery OR repair OR improvement OR healing OR restoration OR effect OR influence OR "aftermath" OR consequence OR impact OR mortality OR morbidity OR "functional recovery" OR "quality of life" OR "return to work"

**Mesh term –** "Patient Outcome Assessment"[Mesh] OR "Outcome and Process Assessment, Health Care"[Mesh] OR "Treatment Outcome"[Mesh]

PUBMED

**(((depression[Title/Abstract] OR "major depressive disorder"[Title/Abstract] OR "Depressive Disorder"[Title/Abstract] OR "common mental disorder"[Title/Abstract] OR "mental disorder"[Title/Abstract] OR "mental health"[Title/Abstract] OR "psychological morbidity"[Title/Abstract] OR "depressive episode"[Title/Abstract] OR "persistent depressive disorder"[Title/Abstract] OR "major depressive disorder"[Title/Abstract]) OR ("Depression"[Mesh] OR "Depressive Disorder"[Mesh] OR "Depressive Disorder, Major"[Mesh]) OR (“Major Depressive Disorder 1" [Supplementary Concept] OR "Major Depressive Disorder 2" [Supplementary Concept])) AND ((stroke[Title/Abstract] OR "hemorrhage stroke"[Title/Abstract] OR "ischemic stroke"[Title/Abstract] OR "transit ischemic stroke"[Title/Abstract]) OR ("Stroke"[Mesh] OR "Stroke, Lacunar"[Mesh] OR "Infarction, Posterior Cerebral Artery"[Mesh] OR "Brain Stem Infarctions"[Mesh] OR "Infarction, Middle Cerebral Artery"[Mesh] OR "Infarction, Anterior Cerebral Artery"[Mesh]))) AND ((outcome[Title/Abstract] OR result[Title/Abstract] OR " end-result"[Title/Abstract] OR product[Title/Abstract] OR rehabilitation[Title/Abstract] OR recovery[Title/Abstract] OR repair[Title/Abstract] OR improvement[Title/Abstract] OR healing[Title/Abstract] OR restoration[Title/Abstract] OR effect[Title/Abstract] OR influence[Title/Abstract] OR "aftermath"[Title/Abstract] OR consequence[Title/Abstract] OR impact[Title/Abstract] OR mortality[Title/Abstract] OR morbidity[Title/Abstract]) OR ("Patient Outcome Assessment"[Mesh] OR "Outcome and Process Assessment, Health Care"[Mesh] OR "Treatment Outcome"[Mesh]))**

Psychinfo

1 exp Atypical Depression/ or exp "Depression (Emotion)"/ or exp Major Depression/ or exp Recurrent Depression/ or exp Treatment Resistant Depression/ or exp Late Life Depression/ 170912

2 exp Cerebrovascular Accidents/ or exp Ischemia/ 28766

3 exp "Treatment Process and Outcome Measures"/ or exp Patient Reported Outcome Measures/ 629

4 (depression or "major depressive disorder" or "Depressive Disorder" or "common mental disorder" or "mental disorder" or "mental health" or "psychological morbidity" or "depressive episode" or "persistent depressive disorder" or "major depressive disorder" or "mood" or "emotional distress").tw. 522121

5 (stroke or "hemorrhage stroke" or "ischemic stroke" or "transit ischemic stroke").tw. 36412

6 (outcome or result or "end-result" or product or rehabilitation or recovery or repair or improvement or healing or restoration or effect or influence or "aftermath" or consequence or impact or mortality or morbidity or "functional recovery" or "quality of life" or "return to work").tw. 1712991

7 1 or 4 534540

8 2 or 5 42287

9 3 or 6 1713067

10 7 and 8 and 9 2873

Search history link psychinfo

<https://access.ovid.com/custom/redirector/wayfless.html?idp=https://kclidpdev.kcl.ac.uk/idp/shibboleth&url=http://ovidsp.ovid.com/ovidweb.cgi?T=JS&NEWS=N&PAGE=main&SHAREDSEARCHID=58dLHUnyaHse4govvw39C0is1t6nIeO4waAJUrQ0GPZvaUhE6KXk9D5l7ulegu3Xc>

https://access.ovid.com/custom/redirector/wayfless.html?idp=https://kclidpdev.kcl.ac.uk/idp/shibboleth&url=http://ovidsp.ovid.com/ovidweb.cgi?T=JS&NEWS=N&PAGE=main&SHAREDSEARCHID=58dLHUnyaHse4govvw39C0is1t6nIeO4waAJUrQ0GPZvaUhE6KXk9D5l7ulegu3Xc

Global medicus index search details

tw:((tw:(depression OR "major depressive disorder" OR "Depressive Disorder" OR "common mental disorder" OR "mental disorder" OR "mental health" OR "psychological morbidity" OR "depressive episode" OR "persistent depressive disorder" OR "major depressive disorder" OR "mood" OR "emotional distress")) AND (tw:(stroke OR "hemorrhage stroke" OR "ischemic stroke" OR "transit ischemic stroke")) AND (tw:(outcome OR result OR "end-result" OR product OR rehabilitation OR recovery OR repair OR improvement OR healing OR restoration OR effect OR influence OR "aftermath" OR consequence OR impact OR mortality OR morbidity OR "functional recovery" OR "quality of life" OR "return to work"))) AND ( fulltext:("1" OR "1") AND type_of_study:("clinical_trials" OR "prognostic_studies" OR "observational_studies") AND la:("en"))

Embase

<https://access.ovid.com/custom/redirector/wayfless.html?idp=https://kclidpdev.kcl.ac.uk/idp/shibboleth&url=http://ovidsp.ovid.com/ovidweb.cgi?T=JS&NEWS=N&PAGE=main&SHAREDSEARCHID=1f5RBU9tJL0QpMj4ojhmYJtIMm9jOvaZ0iPBOpLcEu4NgyjVrnNPUQoxFjjJCD3Hg>

Pubmed

(((depression[Text Word] OR "major depressive disorder"[Text Word] OR "Depressive Disorder"[Text Word] OR "common mental disorder"[Text Word] OR "mental disorder"[Text Word] OR "mental health"[Text Word] OR "psychological morbidity"[Text Word] OR "depressive episode"[Text Word] OR "persistent depressive disorder"[Text Word]) OR ("Depression"[Mesh] OR "Depressive Disorder"[Mesh] OR "Depressive Disorder, Major"[Mesh]) OR ("Major Depressive Disorder 1" [Supplementary Concept] OR "Major Depressive Disorder 2" [Supplementary Concept])) AND ((stroke[Text Word] OR "hemorrhage stroke"[Text Word] OR "ischemic stroke"[Text Word] OR "transit ischemic stroke"[Text Word]) OR ("Stroke"[Mesh] OR "Stroke, Lacunar"[Mesh] OR "Infarction, Posterior Cerebral Artery"[Mesh] OR "Brain Stem Infarctions"[Mesh] OR "Infarction, Middle Cerebral Artery"[Mesh] OR "Infarction, Anterior Cerebral Artery"[Mesh]))) AND ((outcome[Text Word] OR result[Text Word] OR " end-result"[Text Word] OR product[Text Word] OR rehabilitation[Text Word] OR recovery[Text Word] OR repair[Text Word] OR improvement[Text Word] OR healing[Text Word] OR restoration[Text Word] OR effect[Text Word] OR influence[Text Word] OR "aftermath"[Text Word] OR consequence[Text Word] OR impact[Text Word] OR mortality[Text Word] OR morbidity[Text Word] OR "functional recovery"[Text Word] OR "quality of life"[Text Word] OR "return to work"[Text Word]) OR ("Patient Outcome Assessment"[Mesh] OR "Outcome and Process Assessment, Health Care"[Mesh] OR "Treatment Outcome"[Mesh]))
