# Supplementary material for: Impact of depression on stroke outcomes among stroke survivors: Systematic review and meta-analysis
Source: PLoS One. 2023 Dec 1;18(12):e0294668. doi: 10.1371/journal.pone.0294668 (PMC10691726; doi:10.1371/journal.pone.0294668)
Supplement: S2 File — (PDF) [file pone.0294668.s002.pdf]

ADDIS ABABA UNIVERSITY, COLLEGE OF HEALTH SCIENCES (IRB)
አዲስ አበባ ዩኒቨርስቲ ጤና ሳይንስ ኮሌጅ
Institutional Review Board

ANNEX 3
Form AAUMF 03-008

IRB's Decision

Meeting No: 12/2020 Meeting Date: December 30,2020
Protocol number: 120/20/Psy

**Protocol Title:** Depression among stroke survivors and their caregivers: prevalence, course, factors and impact on Rehabilitation outcome in Ethiopia. Mixed methods study			
Principal Investigator:	Sebele Shewangizaw		
Institute:	College of Health Sciences, AAU		
Elements Reviewed (AAUMF 01-008)		☑ Attached	☐ Not attached
Review of Revised Application ☐ Yes ☐ No		Date of Previous review:	
Decision of the meeting:	☑ Approved ☐ Approved with Recommendation ☐ Resubmission ☐ Disapproved		

I. Elements approved-
1. Protocol Version No: 2
2. Protocol Version Date:
3. Informed consent Version No. 2
4. Informed Consent Version Date:

II. Obligations of the PI-
1. Should comply with the standard international & national scientific and ethical guidelines
2. All amendments and changes made in protocol and consent form needs IRB approval
3. The PI should report SAE within 10 days of the event
4. End of the study, including manuscripts and thesis works should be reported to the IRB
5. The PI should report non-compliance and unanticipated events

III. TO NERC ☐

Institution Review Board (IRB) Approval: Period from: April 19, 2021 to April 18, 2022
Follow up report expected in
3 Months ___ 6 months ____ 9 months ✓ one year ____

Chairperson, IRB
Dr. Adamu Addissie

Signature
Date: April 19, 2021

Institutional Review Board Office (IRB) Faculty of Medicine ADDIS ABABA UNIVERSITY

CamScanner
