# Supplementary material for: Impact of depression on stroke outcomes among stroke survivors: Systematic review and meta-analysis
Source: PLoS One. 2023 Dec 1;18(12):e0294668. doi: 10.1371/journal.pone.0294668 (PMC10691726; doi:10.1371/journal.pone.0294668)
Supplement: S1 Table — (DOCX) [file pone.0294668.s003.docx]

Table 2: Quality assessment for impact of depression on stroke outcomes

| **No** | **Author (year)** | **Criteria** | | | | | | **Global rating***** |
| --- | --- | --- | --- | --- | --- | --- | --- | --- |
|  |  | **Selection bias*** | **Study design*** | **Confounders*** | **Blinding*** | **Data collection method*** | **Withdrawals and dropouts**** |  |
|  | Adbdul Sattar et al 2013 | 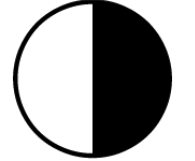 | 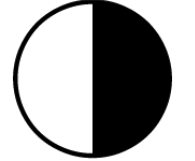 | 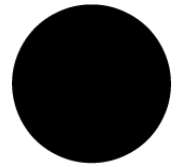 | NA | 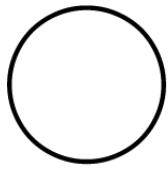 | 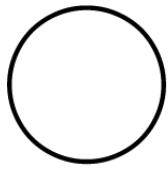 | 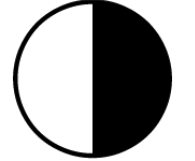 |
|  | Almedia & Xiao et al. 2007 | 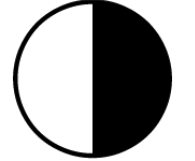 | 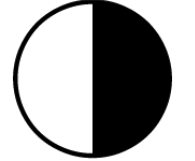 | 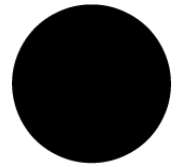 | NA | 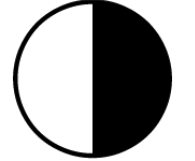 | 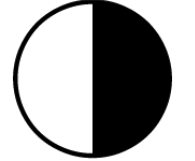 | 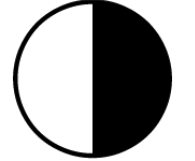 |
|  | Ayreb et al. 2014 | 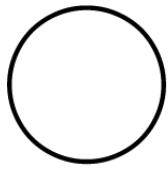 | 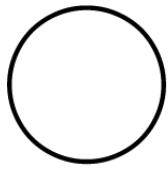 | 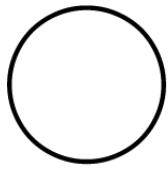 | NA | 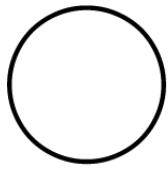 | 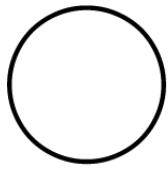 | 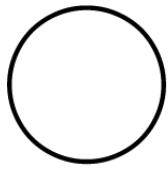 |
|  | Ayreb et al. 2015 | 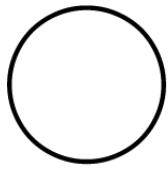 | 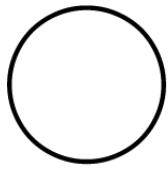 | 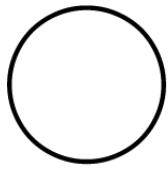 | NA | 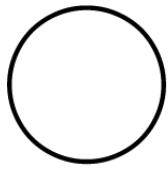 | 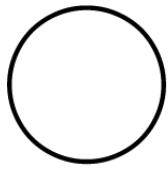 | 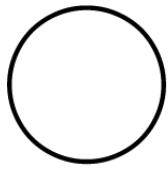 |
|  | Baccaro et al. 2019 | 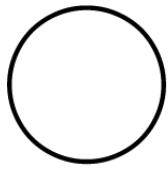 | 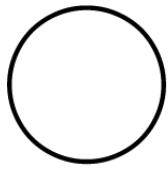 | 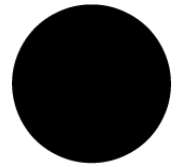 | NA | 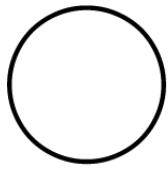 | 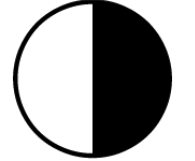 | 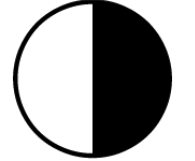 |
|  | Boudokhane et.al 2021 | 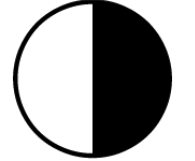 | 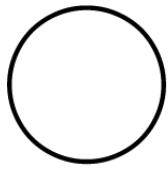 | 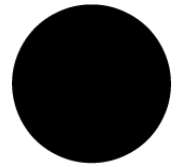 | NA | 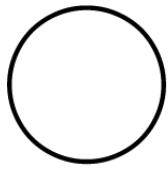 | 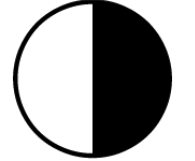 | 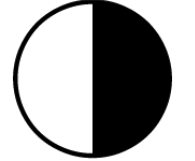 |
|  | Boutros et al. 2022 | 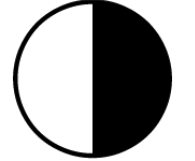 | 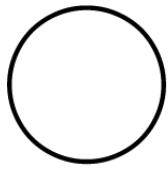 | 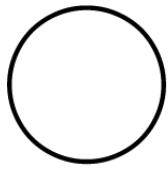 | NA | 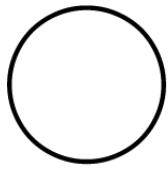 | 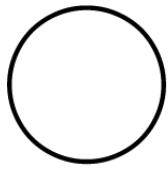 | 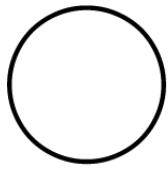 |
|  | Boutros et al. 2023 | 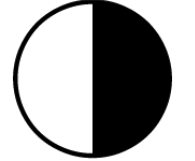 | 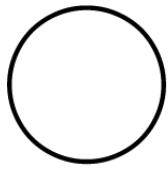 | 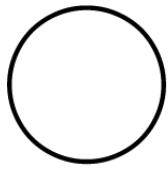 | NA | 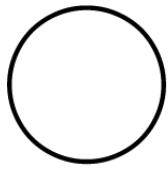 | 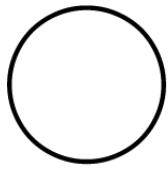 | 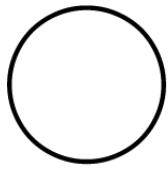 |
|  | Cassidy et al. 2004 | 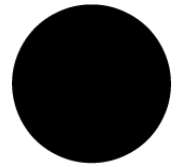 | 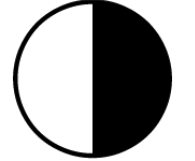 | 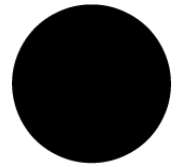 | NA | 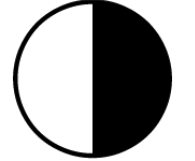 | 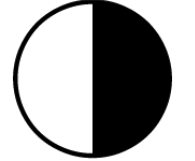 | 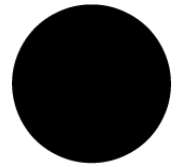 |
|  | Choi et al., 2020 | 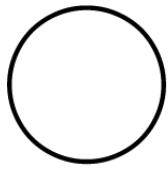 | 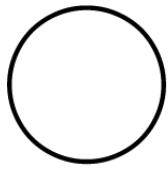 | 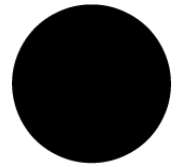 | NA | 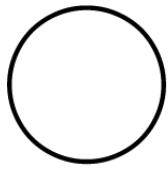 | NA | 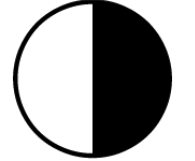 |
|  | Clark et.al 1998 | 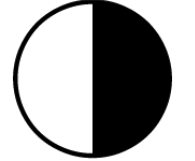 | 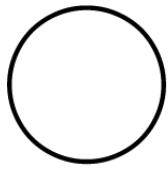 | 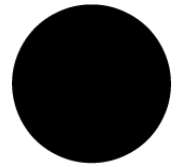 | NA | 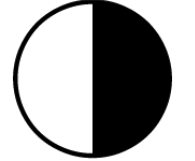 | 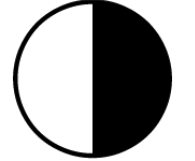 | 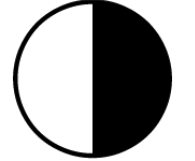 |
|  | De Mello et al. 2016 | 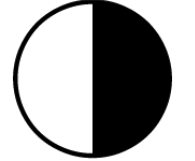 | 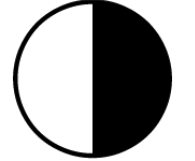 | 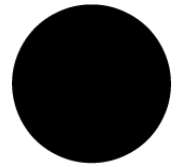 | NA | 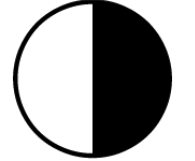 | 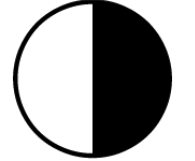 | 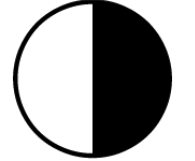 |
|  | Donnellan et al. 2010 | 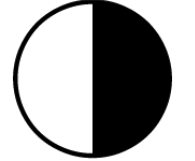 | 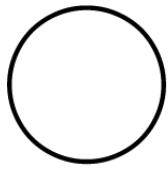 | 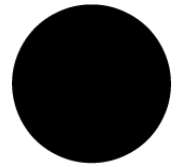 | NA | 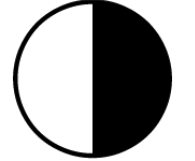 | 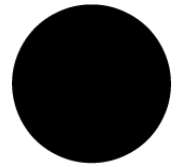 | 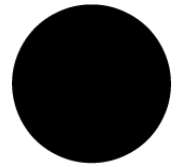 |
|  | El Husseini et al. 2017 | 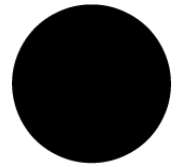 | 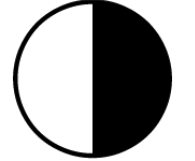 | 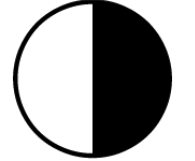 | NA | 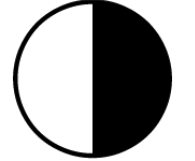 | 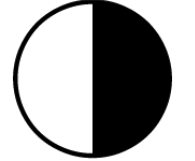 | 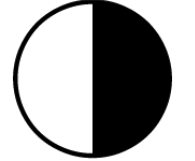 |
|  | Ellis et al. 2010 | 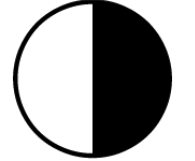 | 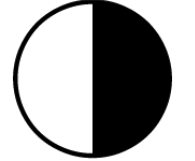 | 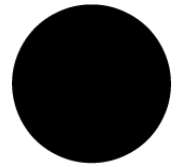 | NA | 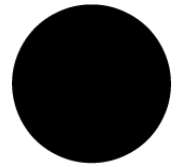 | 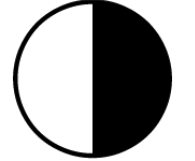 | 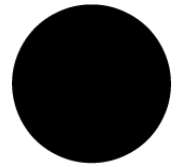 |
|  | Everson et.al 1998 | 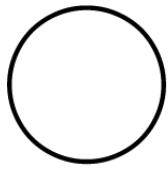 | 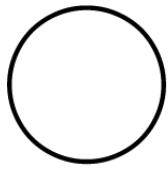 | 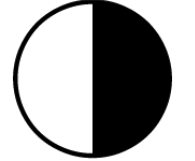 | NA | 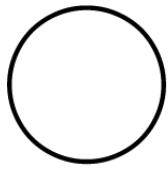 | NA | 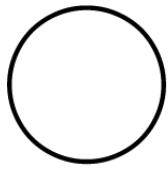 |
|  | Freak-Poli 2018 | 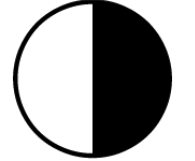 | 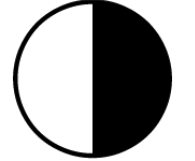 | 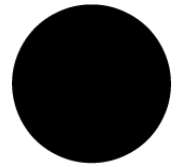 | NA | 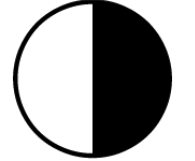 | 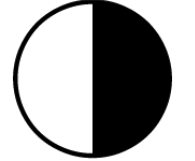 | 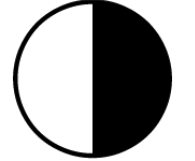 |
|  | Gbril et al. 2010 |  |  |  | NA |  |  |  |
|  | Geun-Young Park et al. 2016 |  |  |  | NA |  |  |  |
|  | Gillen et al. 2001 |  |  |  | NA |  |  |  |
|  | Guajardo et al. 2015 |  |  |  | NA |  |  |  |
|  | Gupta et al. 2022 |  |  |  | NA |  |  |  |
|  | Hackett et al. 2012 |  |  |  |  |  |  |  |
|  | Hama et al. 2001 |  |  |  | NA |  |  |  |
|  | Herrmann et al. 1998 |  |  |  | NA |  |  |  |
|  | Hong et al. 2018 |  |  |  | NA |  |  |  |
|  | Hornsten et al. 2013 |  |  |  | NA |  |  |  |
|  | House et al. 2001 |  |  |  | NA |  |  |  |
|  | Jia et al. 2006 |  |  |  | NA |  |  |  |
|  | Jet van der Kemp 2019 |  |  |  | NA |  |  |  |
|  | Johnston et al. 2004 |  |  |  | NA |  |  |  |
|  | Jorge et al. 2003 |  |  |  | NA |  |  |  |
|  | Jørgensen et al. 2016 |  |  |  | NA |  |  |  |
|  | Kemper et.al. 2011 |  |  |  | NA |  |  |  |
|  | Kang et al. 2018 |  |  |  | NA |  |  |  |
|  | Kauhane et al 1999 |  |  |  | NA |  |  |  |
|  | S. Kijowski 2014 |  |  |  | NA |  |  |  |
|  | Kim et al. 2018 |  |  |  | NA |  |  |  |
|  | Koivisto et al. 1993 |  |  |  | NA |  |  |  |
|  | Kotila et al.1999 |  |  |  | NA |  |  |  |
|  | Kuptniratsaikul et al. 2009 |  |  |  | NA |  |  |  |
|  | Lai et al 2002 |  |  |  | NA |  |  |  |
|  | Lam et al. 2019 |  |  |  | NA |  |  |  |
|  | Li et al. 2019 |  |  |  | NA |  |  |  |
|  | Lin et al. 2020 |  |  |  | NA |  |  |  |
|  | Loong et al. 1995 |  |  |  | NA |  |  |  |
|  | Melkas et al. 2010 |  |  |  | NA |  |  |  |
|  | Matsuzaki et al. 2015 |  |  |  | NA |  |  |  |
|  | Morris et al 1993 |  |  |  | NA |  |  |  |
|  | Morris et al 1993 |  |  |  | NA |  |  |  |
|  | Naess et al. 2010 |  |  |  | NA |  |  |  |
|  | Nannetti et al. 2005 |  |  |  | NA |  |  |  |
|  | Nascimento et.al 2019 |  |  |  | NA |  |  |  |
|  | Novack et al. 1987 |  |  |  | NA |  |  |  |
|  | Orman et al. 2022 |  |  |  |  |  |  |  |
|  | Paolucci et al. 1999 |  |  |  | NA |  |  |  |
|  | Parikh et al. 1990 |  |  |  | NA |  |  |  |
|  | Park et al. 2015 |  |  |  | NA |  |  |  |
|  | Park et al. 2016 |  |  |  | NA |  |  |  |
|  | Pellicciari et al. 2022 |  |  |  | NA |  |  |  |
|  | Pohjasvaara et al. 2001 |  |  |  | NA |  |  |  |
|  | Razmara et al. 2017 |  |  |  | NA |  |  |  |
|  | Ried et al. 2011 |  |  |  | NA |  |  |  |
|  | Saxena et al. 2007 |  |  |  | NA |  |  |  |
|  | Schmid et al. 2011 |  |  |  | NA |  |  |  |
|  | Schubert et al. 1992 |  |  |  | NA |  |  |  |
|  | Schulz et al. 2017 |  |  |  | NA |  |  |  |
|  | Shi et al. 2016 |  |  |  | NA |  |  |  |
|  | Smi et al. 2006 |  |  |  | NA |  |  |  |
|  | Spruit-van Eijk et al. 2012 |  |  |  | NA |  |  |  |
|  | Tse et al. 2019 |  |  |  | NA |  |  |  |
|  | Van de weg et al. 1999 |  |  |  | NA |  |  |  |
|  | Jet van der Kemp et al. 2019 |  |  |  | NA |  |  |  |
|  | Wagle et al. 2011 |  |  |  | NA |  |  |  |
|  | Willey et al. 2010 |  |  |  | NA |  |  |  |
|  | Williams et al. 2004 |  |  |  | NA |  |  |  |
|  | Wilz et al. 2007 |  |  |  | NA |  |  |  |
|  | Wulsin et al 2012 |  |  |  | NA |  |  |  |
|  | Yuan et al. 2014 |  |  |  | NA |  |  |  |
|  | Zikic et al. 2014 |  |  |  | NA |  |  |  |

= Strong (no weak rating) = 13

= Moderate (one weak rating)= 56

= Week (two or more ratings)= 11

NA= Not Applicable
