# Supplementary material for: Impact of depression on stroke outcomes among stroke survivors: Systematic review and meta-analysis
Source: PLoS One. 2023 Dec 1;18(12):e0294668. doi: 10.1371/journal.pone.0294668 (PMC10691726; doi:10.1371/journal.pone.0294668)
Supplement: Attachment — Submitted filename: Response to Reviewers.docx [file pone.0294668.s004.docx]

**Response to reviewers**

We appreciate the comments and suggestions of all the reviewers and editorial requests. We have tried to address the concerns of each reviewer and the editorial requests in the following table.

| **Reviewer** | **Comments** | **Response** |
| --- | --- | --- |
| Editorial requests | Please ensure that your manuscript meets PLOS ONE's style requirements, including those for file naming. | We have reviewed the style requirements and marked our files according to the guideline (response to reviewers, revised manuscript with track changes, clear manuscript, and supplementary files) |
|  | Funding Statement | We have added the recommended statement to the funding statement.  There was no additional external funding received for this study. |
|  | Please review your reference list to ensure that it is complete and correct | We reviewed our reference list and believe it is complete and correct |
| Reviewer: 1  Buna Bhandari | Is the manuscript technically sound, and do the data support the conclusions?  Reviewer #1: No | We strongly commend and hold in high regard the reviewer's decision and expertise. But we strongly believe the manuscript is technically sound, and we have modified the conclusions section |
|  | Has the statistical analysis been performed appropriately and rigorously?  Reviewer #1: No | We have now modified the data analysis section of the meta-analysis. We're hopeful that these changes will help the reviewer reconsider their decision.  Please see the track changes on pages five & six |
|  | Have the authors made all data underlying the findings in their manuscript fully available?  Reviewer #1: Yes | Thanks |
|  | Is the manuscript presented in an intelligible fashion and written in standard English?  Reviewer #1: No | Considering the suggestion of both reviewers, one of the authors Catherine Sackley (professor at the University of Nottingham and a native English speaker) has done editions (please see the important changes across the manuscript) |
|  | This study investigated the impact of depression on stroke outcome which is very important area of research among stoke survivors. I have added my comments for the improvement of quality of the paper.  Title and abstract looks fine. Introduction is presented well with highlighting the gap in literature and there is clarity of research questions. | Thank you. |
|  | Eligibility criteria: It is clear however it’s better to present in sentences ( paragraph) than in a bullet format. | Thank you very much for bringing this to our attention and now we rewrite it as a paragraph.  Please see the track changes on page four  “We included longitudinal studies on post-stroke depression conducted among adults diagnosed with various types of strokes. We included studies that reported both clinically diagnosed depression and studies that utilized screening tools. The outcomes we looked at were functioning, quality of life, returning to work, cognition, and mortality (rate and premature mortality)” |
|  | Data analysis: There are some grammatical errors in writing, Please use past tense. Please mentioned the methods of meta-analysis clearly here and no need of repeating same in the result sections. | Thank you for bringing this to our attention, we have now done a significant change to the methods and result sections of the meta-analysis  Please see the changes on pages five & six |
|  | Study selection process: Please clarify whether you used endnote for the whole screening process or any other SR software such as Revman or Covidence was also used. | Thank you. We have used EndNote for the selection and mentioned this on page five |
|  | PRISMA flow diagram It can be presented in better ways. Please refer this <http://prisma-statement.org/prismastatement/flowdiagram.aspx?AspxAutoDetectCookieSupport=1> and update the diagram accordingly. In addition, I would suggest witting excluded heading above excluded study. | Thank you for the suggestion, we now improved the diagram  Please see page seven |
|  | In addition, I can see reasons of exclusion after full text review are only due to the wrong study design; however usually there would be chances of having other reasons ( wrong study population, wrong outcomes etc.) So please make sure and report if there are any other reasons of exclusion as well. | Thank you for the points. We agree with the reviewer in principle. But because our inclusion criteria are not that strict, we did not exclude studies due to other factors. For example, we included all types of strokes, and we assessed many outcomes |
|  | Please keep the reference of each studies after description such as most of the studies (66 from high income countries ) USA ( keep ref) , England(ref) ……similarly keep reference of the each included study immediately after their description so that reader can locate them easily. | Accepted and tried to cite as suggested. But in some places where we have to cite many papers for a concept, we opt not to do as it takes a line or more.  Please see the track changes from page 8 to 18 |
|  | Same comments applies to other places as well such as 4 studies assessed the PSD – keep ref for those study immediately after text. Same goes to - Risk of Bias section - keep references of studies with strong, moderate and weak quality. | Accepted and corrected.  Please see the track changes from page 8 to 18 |
|  | Inside table 1 as well , keep ref after each study. The length of follow up in each studies varies significantly such as from 28 days up to 10 year, I was wondering whether such variation of study duration has some effects on the outcome or not? why the length of follow up was not considered during deciding the inclusion criteria of the studies? Please briefly clarify your approach. | These are important points. The length of follow-up varies as mentioned. These significantly affect the result and we now mentioned this in the limitation. One of our aim was to compare the short and the long-term impact but the variations in measures and other parameters restricted us from doing so. |
|  | Same comments, author has not cited the references properly to locate the study in most of the places such as second para under impact of depression on cognitions. | Corrected.  Please see the track changes from page 8 to 18 |
|  | Please apply above comment in each places whenever you refer any included studies, keep the reference immediately after that to locate them easily.  Language editing of the manuscript is advised. | Corrected.  Please see the track changes from page 8 to 18 and the discussion section. |
|  | Meta-analysis The method of meta analysis can be included in the method section than in the result. Authors has not interpreted the meta analysis finding properly for example in text of Fig 2 only. The effect size was 1.94 with a 95% confidence interval of 1.38 to 2.73 is mentioned. But you should report what does 1.94 mean in this case so that reader can get the result correctly and similarly interpret and report each forest plot finding accordingly. I would suggest writing the descriptive result concisely than repeating and putting findings here and there. For e.g., descriptive result and meta-analysis of same result ( such as PSD and cognition) can be kept together and interpret accordingly rather than keeping all the forest plot separately in the text. In addition, range of OR in forest plot is very wide 1, 10 and 100… please try to revise it. In addition, significance of the findings presented in the forest plot is not interpreted these finding accurately for e.g. what is the difference in finding of fig 1-3 and 4, what does those non-significant findings of fig 4 and significance in other figures means. Please take care of everything and report them clearly. In addition, what about the quality of all the included studies? whether some of the studies with strong evidence and some with weak were analyzed together or not in meta-analysis ? as only homogenous study should be included in the meta-analysis. If that has any effect in the pooled result or not. Please clarify | Accepting the comment we now addressed it. Please see pages five & six  Eleven studies reported the impact of depression on functioning. Stroke survivors with depression reported higher functioning problems than stroke survivors without depression(pooled OR =1.94; 1.38, 2.73)… |
|  | Discussion This section needs major revision. The comparison with previous evidences and critical interpretation and argument is lacking in this section. I would advise rather than repeating what is mention in the result section, please try to interpret the findings and present possible reasons and ways forward with the support of evidences. In addition, there is plenty of grammatical and typos errors throughout the manuscript. Even the spelling of meta-analysis is spelled as "metanalysis" in multiple places. Please double check. | Accepting the recommendation, we now revised the discussion section. Please see the track changes from page 25 to 28 |
|  | Conclusion Conclusion is not presented strongly. Please don’t write basic and general conclusion, it should be based on your study findings. Usually SRM evidence are considered strong for the future policy and program but I can’t see any special recommendation from this SRM. Please clarify.  Overall good attempt by the authors. But this manuscript need major language editing and revision of content and process to improve the quality of the paper. | We also revise the conclusion as  “Depression affects many aspects of stroke outcomes including survival. The evidence is not conclusive on some outcomes such as cognition. This review indicated the need for longitudinal studies with higher sample size especially in low-resource settings since the treatment for depression and stroke is not well-established and there is no well-established social welfare system. It also showed the need to provide mental health support for stroke survivors as it is related to better overall health and recovery.” |
| Reviewer: 2 | The authors present a systematic review and meta-analysis on “Impact of depression on stroke outcomes among stroke survivors.” This is an important area and I congratulate the authors on coming up with this piece of writing. I advise the authors to further update if any further reviews or prospective studies have been conducted meanwhile. | Our appreciation also goes for this reviewer. We appreciate the points raised. Based on the suggestion, we have updated the review but we didn’t find a study that fulfils the inclusion criteria. |
|  | Title: The title appears misleading if the authors are looking at the effects of post- stroke depression. Depression in stroke patients does not always appear post- stroke. Those might have had depressive diagnosis before having stroke. So, I request them to clarify. Please clarify this issue throughout the manuscript at all instances. | It is an important point. We aimed to see the impact of post-stroke depression on stroke outcomes. As mentioned, depression may happen before the stroke and it is difficult to say which comes first. Due to the nature of the illness (as some may not seek help and get their diagnosis) we fail to say it is a post-stroke depression. That is why we opt not to specify it in the title. We have now included this as a possible limitation of the review. |
|  | Introduction: The second sentence requires paraphrasing in the first paragraph. In second paragraph, please be specific regarding post- stroke depression. When you say post-stroke depression, do you have any operational definition for post stroke depression? Please clarify because this is the main theme for your SR. Also, mention briefly why post- stroke depression is important from theoretical perspective and its clinical relevance. You can also give prevalence estimates from updated reviews. In the background information where the authors cite multiple reviews, what do those reviews tell about the effects of post stroke depression? Do all the reviews mention regarding post stroke depression in stroke survivors? | Corrected based on the comments. Please see the track changes on page three  We define PSD. Please see the track changes on page three  We have revised the results of the previous reviews and indicated the gap. Please see the track changes on page three |
|  | Eligibility criteria: What is the operational definition of post stroke depression? There are many outcomes in the study, and they are quite broad. Could this be a limitation to the study? For example, when you say cognition, it denotes many things. What were the authors referring to when they mention cognition? | We have now defined PSD. Please see the changes on page three  We included longitudinal studies on depression conducted among adults diagnosed with various types of strokes. We included studies that reported both clinically diagnosed depression and studies that utilized screening tools. Some studies exclude survivors with known depression while others do separate analyses.  The outcomes are broad as mentioned. It had both limitations and advantages. It restricts us from giving details but also gives us the chance to report the multidimensional impacts. We understand the different dimensions of cognition and we included studies that report any aspect of it. |
|  | Did you have any exclusion criteria? Did you come across situations when it was tough to decide to include or exclude a study based on your eligibility? | We didn’t put any exclusion criteria. There were situations where deciding to include/exclude was difficult. When these situations happen, the investigators bring them to the research team. |
|  | Study selection process: Who was the third reviewer involved in discrepancy resolving? Was there any discrepancy? | One of the investigators (YG) was involved in solving discrepancies. We now mentioned this. Please see the track changes on page five |
|  | The authors do not mention if risk of bias assessment was done though quality check was done. Please clarify and what tool was used? To me, it appears there was overlap between the two terms in the manuscript. | We have used the Effective Public Health Practice Project (EPHPP) to assess the quality of the evidence. The tool assesses the possible biases including selection bias, assessment of confounding and other sources of biases. |
|  | Results: Tables  I wonder if all the studies included are prospective. There is great heterogeneity in the studies included. I also see from table 1 that in some studies, e.g., Gbiri et al. 2010., depression at onset is mentioned. What does this mean? In another study by Guajardo [et.al](http://et.al/). 2015. and Donellan et al. 2010. , there is mention of depressive symptoms, rather than diagnosis of depression. Depressive symptoms are common in acute stages post-depression, but depression diagnosis deserves clinical attention for various reasons. Please clarify. | Agreeing the heterogeneity of the studies, all the studies are longitudinal. We now edited some wording such as onset to “baseline” to avoid such confusion. Since the studies used various measures and most of them are screening measures we decided to include both clinically diagnosed depression and studies that used screening measures. |
|  | The authors are further advised to mention the setting of the studies besides the country such as outpatient, inpatient, physiotherapy etc. and also the title of the studies in the table itself.  Same goes for other tables. Please clarify. | We appreciate the comment. However, we think stating the specific setting and the title will not add anything to the reader because we reported if there was treatment for depression and the duration of the illness. We now add citations to each outcome instead of adding a citation to the tables. |
|  | Meta- analysis: Please provide a clear rationale for inclusion of specific studies. What was your aim to do a meta- analysis before planning this? The authors are also advised to provide inferences about the reported results. Did you measure variance? Please explain. | We now modify the meta-analysis sections in the methods and the result. Please see pages five & six, 23 & 24 |
|  | There are other issues as well. Some methodology statements are described in results section. Please consider keeping them where suited best. | Accepted and corrected. |
|  | Discussion: This section needs major rewriting based on all the clarifications after comments. Further, the authors are advised to discuss based on the reported findings and literature available. I believe there are other biases and limitations besides those mentioned. Please elaborate on those. | We have updated the discussion based on the suggestions  Please see the track changes from pages 25 to 28 |
|  | Minor issues:  There are many grammatical and typo errors throughout the manuscript. Also, I see that the supplementary files are not correctly named and not placed sequentially in the texts. Please correct them according to journal’s policy. | Considering the suggestion of both reviewers, one of the authors Catherine Sackley (Professor at the University of Nottingham and a native English speaker) has done editions (please see the important changes across the manuscript) |
