# Supplementary material for: Impact of depression on stroke outcomes among stroke survivors: Systematic review and meta-analysis
Source: PLoS One. 2023 Dec 1;18(12):e0294668. doi: 10.1371/journal.pone.0294668 (PMC10691726; doi:10.1371/journal.pone.0294668)
Supplement: Attachment — Submitted filename: Response to Reviewers.docx [file pone.0294668.s005.docx]

**Response to reviewers**

We appreciate the reviewers and the editor for the comments and suggestions and for recognizing the updates we made. We have tried to address the current concerns and the editorial issues. Please see the details in the following table and the track changes in the manuscript.

| **Reviewers** | **Number** | **Comments** | **Response** |
| --- | --- | --- | --- |
| Editorial |  | Please review your reference list to ensure that it is complete and correct. If you have cited papers that have been retracted, please include the rationale for doing so in the manuscript text, or remove these references and replace them with relevant current references. Any changes to the reference list should be mentioned in the rebuttal letter that accompanies your revised manuscript. If you need to cite a retracted article, indicate the article’s retracted status in the References list and also include a citation and full reference for the retraction notice. | We have tried to adhere to PLOS referencing guidelines and avoid citing retracted articles. We have edited the reference for stroke symptoms in the introduction. We have also made edits to the reference for the quality assessment checklist because it didn't align with the guidelines set forth by the journal. |
| Reviewers’ comments | 1 | Abstract- data extraction- please write two authors extracted data from the studies than writing "extracted the studies" | Thank you very much for the suggestion. We have now modified it as suggested. |
|  | 2 | You can provide PROSPERO registration number below abstract than within abstract | Accepted and modified. Please see the track changes |
|  | 3 | Better to write abstract accordingly to PLOS one Standard format - Intro, Methods, Results and conclusions | Thank you for this suggestion. However, we believe the current format makes it easier to read the abstract. We thought the recommended style suited a report from a primary study. |
|  | 4 | Result section stills lacks adequate referencing to the respective articles especially in the characteristics of the included study and ROB section. It would be difficult to locate included studies if it is not clearly referred within text. | Thank you again for raising this important point. We have added citations in the result section. However, we didn't cite the ROB section, because the list is quite lengthy. Please also note that we have cited a supplementary file for this section. |
|  | 5 | In addition, number of the articles are edited in the revised PRISMA diagram, I was wondering about the reason. I assume author did not run search again after submission of the first manuscript. In addition, it is not appropriate to write hand search - 2 in the full text review section in the PRISMA diagram. Was those 2 article were directly included for full text review without doing title and abstract screening. Not clear to me. | Thank you very much for this feedback. It had been more than six months since we did the search when we resubmitted the manuscript, we then ran the search again and brought up-to-date information. That is why we edited the number. The two articles were directly added to the full-text review because we found them in the reference of the articles included in the full-text (forward and backward search). We believe this is one strength of our review. |
|  | 6 | I still preferred to have different heading for the result of meta-analysis rather than keeping meta-analysis as a subheading. It is a statistical methods not the content of result. | Thank you again. We have now modified the heading as  “Pooled impact of depression on functioning and mortality rate” |
|  | 7 | Discussion and conclusion are improved than before. | Thank you. |
|  | 8 | References are not uniform fonts style as manuscript. Please follow the journal guidelines for formatting the article. | Accepted and modified. Please see the track changes |
