# Supplementary material for: Impact of depression on stroke outcomes among stroke survivors: Systematic review and meta-analysis
Source: PLoS One. 2023 Dec 1;18(12):e0294668. doi: 10.1371/journal.pone.0294668 (PMC10691726; doi:10.1371/journal.pone.0294668)
Supplement: Attachment — Submitted filename: Response to Reviewers3.docx [file pone.0294668.s006.docx]

**Response to reviewers**

We appreciate the reviewer and the editor for the comments and suggestions and for recognizing the updates we made. We have tried to address the current concerns and the editorial issues. Please see the details in the following table and the track changes in the manuscript.

| **Reviewer** | **Number** | **Comments** | **Response** |
| --- | --- | --- | --- |
| Editorial | 1 | Please review your reference list to ensure that it is complete and correct. If you have cited papers that have been retracted, please include the rationale for doing so in the manuscript text, or remove these references and replace them with relevant current references. Any changes to the reference list should be mentioned in the rebuttal letter that accompanies your revised manuscript. If you need to cite a retracted article, indicate the article’s retracted status in the References list and also include a citation and full reference for the retraction notice. | We have tried to adhere to PLOS referencing guidelines and avoid citing retracted articles. We have edited the reference for stroke symptoms in the introduction. We have also made edits to the reference for the quality assessment checklist because it didn't align with the guidelines set forth by the journal. |
| Reviewer’s comments | 1 | Thank you for addressing most of the comments. Manuscript is improved a lot.  However, I still have some concern.  Format of abstract- Though it is systematic review, without background (showing gap in the literature), directly keeping objectives does not provide enough information for the reader.  In addition, data source, eligibility, data analysis all comes under the methods section of SRM. So, it is advisable to include them under methods. | Thank you very much for noticing the changes we made based on your previous suggestions.  We have tried to incorporate this suggestion-indicating the research gap and our objective in this review.  Please see the track changes on page 2 |
|  | 2 | Secondly, I still don't see significance of keeping Hand search (2 article) in full text section directly in PRISMA flow diagram. Because, if your search strategy is not comprehensive and could not search any previously published studies then hand-search is a way to ensure all the eligible study is included in your Review. | We have also tried to accommodate this suggestion and modified the numbers.  Please see the track changes on the PRISMA diagram |
